# A New Application of Electricity

**Published:** 1865-07

**Authors:** 


### A NEW APPLICATION OF ELECTRICITY.

EDITOR REGISTER:—In the May Number of the DENTAL REGISTER, (1864), I notice an article from the "*American Medical Times.*" Giving (as he supposed) a description of a *new* discovery, or application of electricity. Without further remarks let me inform "Mr. Robt. Cornelius, that if he had read the Patent Office Report of 1859, page 221, No. 22,9 2, he would have saved himself a great deal of trouble, labor and perhaps some expense. W. W. Hopkins, of Amelia, Ohio, is the discoverer of the new application claimed by Mr. Cornelius, and so far tested it, that he says he can light up a whole city at once with a single battery. He proposed to Mr. Pike, of Cincinnati, to light up his Opera-House with the battery. and was at one time about perfecting an arrangement to that effect. Why he did not, I do not know. He has used upwards of five thousand feet of wire and says the same spark will light as well at the farthest end as it will at any other point.

So far as the benefits of such a discovery are concerned, they are great, and the only wonder to me is that our City Council has not taken action in the matter. The expense of lighting the gas in one of our large cities is enormous, and might all be saved by the use of the electrical current.

Mr. Hopkins is extremely anxious to have something done with his Patent, but don't feel able to bear the expense of it. He will dispose of City privileges on *very reasonable terms.* The apparatus is so simple that it may be used in families, societies, churches, hotels, or any where that gas is used.

He has made a valuable improvement since the Patent was issued, which is, or will be covered by Letters Patent.

I make this statement simply that the right person will have the glory. W. H. S.

